# Sphingolipid changes in mouse brain and plasma after mild traumatic brain injury at the acute phases

**DOI:** 10.1186/s12944-024-02186-x

**Published:** 2024-06-27

**Authors:** Koushik Mondal, Nobel A. Del Mar, Ashlyn A. Gary, Richard C. Grambergs, Mohd Yousuf, Faiza Tahia, Benjamin Stephenson, Daniel J. Stephenson, Charles E. Chalfant, Anton Reiner, Nawajes Mandal

**Affiliations:** 1 Department of Ophthalmology, The University of Tennessee Health Science Centre, Memphis, TN 38163 USA; 2 Miller School of Medicine, University of Miami, Miami, FL USA; 3 Departments of Medicine and Cell Biology, University of Virginia School of Medicine, Charlottesville, VA 22903 USA; 4 Research Service, Richmond VA Medical Center, Richmond, VA 23298 USA; 5 Department of Anatomy and Neurobiology, The University of Tennessee Health Science Centre, Memphis, TN 38163 USA; 6 Department of Pharmaceutical Sciences, College of Pharmacy, The University of Tennessee Health Science Centre, Memphis, TN 38163 USA; 7 Memphis VA Medical Center, Memphis, TN 38104 USA; 8 Molecular Diagnostics Laboratory, Department of Basic & Translational Research, Saroj Gupta Cancer Centre & Research Institute, Kolkata, WB 700 063 India

**Keywords:** Traumatic brain injury, Sphingolipid, Ceramide, Sphingomyelin, Sphingomyelinase, Inflammation

## Abstract

**Background:**

Traumatic brain injury (TBI) causes neuroinflammation and can lead to long-term neurological dysfunction, even in cases of mild TBI (mTBI). Despite the substantial burden of this disease, the management of TBI is precluded by an incomplete understanding of its cellular mechanisms. Sphingolipids (SPL) and their metabolites have emerged as key orchestrators of biological processes related to tissue injury, neuroinflammation, and inflammation resolution. No study so far has investigated comprehensive sphingolipid profile changes immediately following TBI in animal models or human cases. In this study, sphingolipid metabolite composition was examined during the acute phases in brain tissue and plasma of mice following mTBI.

**Methods:**

Wildtype mice were exposed to air-blast-mediated mTBI, with blast exposure set at 50-psi on the left cranium and 0-psi designated as Sham. Sphingolipid profile was analyzed in brain tissue and plasma during the acute phases of 1, 3, and 7 days post-TBI via liquid-chromatography-mass spectrometry. Simultaneously, gene expression of sphingolipid metabolic markers within brain tissue was analyzed using quantitative reverse transcription-polymerase chain reaction. Significance (*P-values*) was determined by non-parametric *t*-test (Mann–Whitney test) and by Tukey’s correction for multiple comparisons.

**Results:**

In post-TBI brain tissue, there was a significant elevation of 1) acid sphingomyelinase (aSMase) at 1- and 3-days, 2) neutral sphingomyelinase (nSMase) at 7-days, 3) ceramide-1-phosphate levels at 1 day, and 4) monohexosylceramide (MHC) and sphingosine at 7-days. Among individual species, the study found an increase in C18:0 and a decrease in C24:1 ceramides (Cer) at 1 day; an increase in C20:0 MHC at 3 days; decrease in MHC C18:0 and increase in MHC C24:1, sphingomyelins (SM) C18:0, and C24:0 at 7 days. Moreover, many sphingolipid metabolic genes were elevated at 1 day, followed by a reduction at 3 days and an absence at 7-days post-TBI. In post-TBI plasma, there was 1) a significant reduction in Cer and MHC C22:0, and an increase in MHC C16:0 at 1 day; 2) a very significant increase in long-chain Cer C24:1 accompanied by significant decreases in Cer C24:0 and C22:0 in MHC and SM at 3 days; and 3) a significant increase of C22:0 in all classes of SPL (Cer, MHC and SM) as well as a decrease in Cer C24:1, MHC C24:1 and MHC C24:0 at 7 days.

**Conclusions:**

Alterations in sphingolipid metabolite composition, particularly sphingomyelinases and short-chain ceramides, may contribute to the induction and regulation of neuroinflammatory events in the early stages of TBI, suggesting potential targets for novel diagnostic, prognostic, and therapeutic strategies in the future.

**Supplementary Information:**

The online version contains supplementary material available at 10.1186/s12944-024-02186-x.

## Introduction

Traumatic brain injury (TBI) is a leading cause of death and disability worldwide, impacting approximately 69 million individuals annually [1]. Among those who survive a TBI, consequences span a spectrum of several short and long-term impacts, including cognitive impairment, sensory abnormalities in hearing and sight, onset of psychiatric disorders, and increased susceptibility to neurodegenerative disorders such as Parkinson’s disease and Alzheimer’s disease [2–6]. Despite the substantial burden of this disease, diagnosis and treatment of TBI are limited by an incomplete understanding of its pathophysiology [7–9]. TBI involves structural damage to the brain parenchyma and vasculature occurring secondary to mechanical forces such as blunt impacts, rapid accelerations, or explosive blasts, resulting in acute neurological dysfunction, which may lead to permanent disability or death [10–13]. Transmission of these forces through solid tissue-liquid interfaces and areas of high tissue density variation between gray and white matter can cause microvascular compromise and diffuse axonal injury, contributing to acute neurological dysfunction in the acute injury phase of TBI. Secondary brain injury may occur due to subsequent disruption of brain homeostatic mechanisms, activation of inflammatory cascades, disruption of cerebral blood flow, excitotoxicity, and cerebral metabolic dysfunction [8, 9, 13, 14]. The persistent activation of secondary neuroinflammatory pathways may cause chronic, progressive neurodegeneration and neurological dysfunction in the long term, even in cases of mild TBI [15–19].

In 1974, the Glasgow Coma Scale (GCS) was designed as an injury severity score to predict outcomes following TBI [20]. Evaluating motor, eye, and verbal responses, the GCS is one of the most widely used clinical tools to assess the extent of impaired consciousness [21]. However, the GCS demonstrates limited predictive value of outcomes in patients with mild-to-moderately severe TBI [22, 23]. Moreover, while imaging modalities such as magnetic resonance imaging (MRI) and computer tomography (CT) are frequently employed to provide clinicians with more objective information, these also often lack sensitivity in detecting mild-to-moderate brain injury [24]. Thus, there is a strong clinical need to develop biomarkers with high specificity and sensitivity to enhance the precision of diagnosing and predicting outcomes in TBI. Over the last decade, this need has prompted research to identify a diverse range of biomarkers [25–28]. Among these biomarkers, there is a relatively limited representation of lipids, although lipids are highly abundant in the brain, constituting more than 50% of its dry weight [29].

Lipids are critical contributors to both physiology and pathology in the central nervous system (CNS) [30–33]. They serve as structural components of cell membranes, function as energy storehouses, and act as important signaling molecules [34]. Given the relatively high concentrations of bioactive lipids, lipid precursors, and metabolites within the CNS, tight regulation of lipid metabolism is crucial for maintaining homeostasis in the brain and reacting to injury or other insults [35]. Sphingolipids (SPL), a significant component of CNS lipids, have emerged as integral mediators of biological processes relating to tissue injury, neuroinflammation, and resolution of inflammation [36]. Though a relatively minor component of total cellular lipids, sphingolipids have emerged as one of the most pathogenically associated lipids, and abnormal sphingolipid metabolism has been linked with numerous neurodegenerative and psychiatric diseases in the literature [37–44]. Recent advances in lipidomic technology have enabled the measurement of individual sphingolipid species within the brain, blood, and cerebrospinal fluid with high accuracy [45–47].

Plasma SPL has been investigated as a biomarker for neurodegeneration, neuroinflammation, and psychiatric diseases; cardiovascular and coronary diseases; in various types of cancers; and for type 1 and type 2 diabetes [37–44]. In addition, SPL markers are being developed as a next-generation “cholesterol” for human cardiac diseases, and testing of plasma ceramides has begun being utilized as a diagnostic marker for cardiac myopathy (MI-HEART ceramides) by the Mayo Clinic. Alterations in plasma SPL profiles have been documented in models of TBI and cerebral ischemia [48–50]. Over the past decade, our research team has developed a mouse model of TBI, thoroughly characterizing its pathology to closely mirror mild TBI in humans [51]. Recent studies revealed significant elevations in ceramide levels in the brains of mice one month following exposure to blast-induced TBI [52]. Findings such as these lend support to the theory that regulation of SPL metabolism and signaling may be critical pathways of TBI pathophysiology. However, their extreme diversity in structure, metabolism, and function necessitates a deeper understanding of the association between specific sphingolipid profiles with specific disease pathology. Currently, there is little information regarding changes in SPL profiles in the timeframe immediately following exposure to TBI. The present study adds novel information to the literature by analyzing the SPL profiles in both the brains and plasma of mice exposed to mild TBI, activity of major SPL metabolic enzymes, and gene expression of SPL enzymes at 1, 3, and 7 days post-TBI.

## Materials and methods

### Animals and TBI methods

All animal handling protocols and experiments were performed according to the rules and regulations of the Association for Research in Vision and Ophthalmology Statement for the Use of Animals in Ophthalmic and Vision Research, the National Institutes of Health (NIH), the Society for Neuroscience, and the University of Tennessee Health Science Center Guidelines for Animals in Research. Euthanasia, tissue harvesting, and other protocols were approved by the UTHSC Institutional Animal Care and Use Committee (UTHSC IACUC). C57BL/6 J mice, used as wild type (WT) control mice were born and raised in the UTHSC LACU (Laboratory Animal Care Unit) vivarium and maintained from birth under cyclic light (50–100 lx, 12 h. on/off, 7 a.m. to 7 p.m. CST). The parents and the offspring were on a regular mouse chow diet.

All the experimental mice were exposed to an air blast to generate the mild TBI as per earlier published protocol [51, 53]. A modified paintball gun was used by a small horizontally mounted air canon system to generate the mild TBI (Invert Mini, Empire Paintball, Sewell, New Jersey, USA). A 0-psi blast was referred to as “sham blast,” and all experimental outcomes were compared between sham and 50-psi blasts. Mice were anesthetized with Avertin (400 mg/kg body weight) and exposed to the blast on the left side of the cranium between ear and eye. Before the blast, the targeted head region was shaved, and a white dot was painted in the middle of the region. A foam rubber sleeve was used to place the animal inside a Plexiglass tube in such a way the targeted head region was positioned in the center of a 7.5 mm diameter hole of the tube, facing the blast cannon barrel tip. The system is arranged to expose the parietal region of the mouse head between the ear and eye to be blasted with specific air pressure, which is set by the transducer and analyzed by a LabVIEW software (National Instruments, Austin, Texas, USA).

At the same h post-TBI (day 1, day 3, or day 7), the mice were sacrificed, and the brain tissue was immediately harvested and snap-frozen in liquid nitrogen and stored at -80 °C. The frozen brains were thawed in ice and homogenized with ice-cold RNAse-free phosphate buffer, fractionated in five parts, snap-frozen in liquid N2, and stored back to -80 °C until used for analysis. Whole blood was immediately collected in EDTA-containing (anti-coagulant) microcentrifuge tubes and placed on ice. The blood sample was then centrifuged at 4 °C within 1 h of collection, and the plasma was isolated from the particulate matter and snap-frozen in liquid N2 and stored at -80 °C until analysis.

#### Sphingomyelinase assay

The entire brain of a mouse was homogenized in RNAse-free phosphate buffer and divided into 5 fractions. One of the fractions was then used to isolate proteins and quantify them using a bicinchoninic acid assay. Neutral and Acidic sphingomyelinase (nSMase, and aSMase, respectively) activity was measured from isolated protein samples from mouse brain tissue of Sham and Blast-exposed mice harvested a 1, 3 and 7 days post-TBI by using Amplex Red Sphingomyelinase Assay kit (Thermo Fisher, Waltham, Massachusetts, USA) as previously described [54].

### Sphingolipids analysis: sphingolipid preparation

Plasma (50 uL) was extracted using a modified Bligh and Dyer Extraction as previously described [55–61]. Samples were spiked with 250 pmol of C1P, sphingomyelin, ceramide, and monohexosyl ceramide (d18:1/12:0 species), and sphingansine (So), sphinganine (Sa), sphingosine-1-phosphate (S1P), sphinganine-1-phosphate (Sa1P) (d17:0 sphinganine/d17:1 sphingosine) as internal standard (Avanti Polar Lipids, Alabaster, Alabama, USA). Following addition of IS, 3 mL of MeOH:CHCl_3_ (2:1) was added to the plasma and the mixture was sonicated to disperse plasma clumps. Samples were then incubated for 6 h at 48 °C. Extracts were then centrifuged at 5000 rpm for 20 min, transferred to a new glass tube, dried down and reconstituted in methanol (500 uL) by sonicating. Extracts were again centrifuged at 5000 rpm for 20 min and transferred to injection vials for mass spec analysis. Tissue samples (ranging from 4.6–48.5 mg in weight) were extracted by transferring sample tissue to tubes with ceramic beads and a 1 mL solution of 50:50 Methanol:Water with 250 pmol of the above described sphingolipid standard. Samples were then homogenized via Bead Rupter Elite (Omni International, Miami, Florida, USA) for 5 min per samples. The entire homogenate was transferred to glass borosilicate tubes and 3 mL of MeOH:CHCl_3_ (2:1) was added to the tissue. The rest of the extraction was carried identically to the plasma as described previously and final tissue concentrations were normalized by their weights.

## Liquid chromatography/mass spectrometry

Sphingolipids were separated using a Shimadzu Nexera X2 LC-30AD coupled to a SIL-30AC auto injector, coupled to a DGU-20A5R degassing unit in the following way. An 8 min, reversed phase LC method utilizing an Acentis Express C18 column (5 cm × 2.1 mm, 2.7 µm) was used to separate the eicosanoids at a 0.5 mL/min flow rate at 60 °C. The column was equilibrated with 100% Solvent A [methanol:water:formic acid (58:44:1, v/v/v) with 5 mM ammonium formate] for 5 min and then 10 µl of sample was injected. 100% Solvent A was used for the first 0.5 min of elution. Solvent B [methanol:formic acid (99:1, v/v) with 5 mM ammonium formate] was increased in a linear gradient to 100% Solvent B from 0.5 min to 3.5 min. Solvent B was held constant at 100% from 3.5 min to 6 min. From 6 min to 6.1 min solvent B was reduced to 0%, and solvent A returned to 100%. Solvent A was held constant at 100% from 6.1 min to 8 min. Sphingolipids were analyzed via mass spec using an AB Sciex Triple Quad 5500 Mass Spectrometer. Q1 and Q3 were set to detect distinctive precursor and product ion pairs. Ions were fragmented in Q2 using N2 gas for collisionally induced dissociation. Analysis used multiple-reaction monitoring in positive-ion mode. Sphingolipids were monitored using precursor → product MRM pairs. The mass spectrometer parameters used were: Curtain Gas: 30 psi; CAD: Medium; Ion Spray Voltage: 5500 V; Temperature: 500 °C; Gas 1: 60 psi; Gas 2: 40 psi; Declustering Potential, Collision Energy, and Cell Exit Potential vary per transition. The species of sphingomyelin (SM); ceramide (Cer), hexosyl-ceramide (Hex-Cer), sphingoid lipids such as sphingosine (Sph); DHS1P dihydro-sphingosine (DH-Sph), and S1P were identified based on their retention time and mass-to-charge (*m/z)* ratio, and quantified as described in previous publications [62]. In this targeted analysis, species of sphingolipids are measured semi-quantitatively in pmols by comparing their peak area values with the internal standards (which are used at 250 pmol/ sample). Mole % comparison is the most common way of comparing two or many groups of samples in which picomol values of each species are converted to the % of total picomol of that particular group. For example, the Mole % of Cer C18:0 = (pmol of Cer C18:0)/ (pmol total Cer) × 100 [63, 64].

### mRNA analysis by RT-PCR

Total RNA was extracted from one of the fractions of the mouse brain using a Life Technologies RNA extraction kit (Sigma-Aldrich Inc. St. Louis, Missouri, USA), and quantitative RT-PCR was performed as previously reported protocol [65]. Then mRNA level was measured for the sphingolipid metabolic genes that include *Serine palmitoyl transferase 1* (*Spt1*) and *Spt2; Sphingosine kinase 1* (*Sphk1*)*,* and *Sphk2; Ceramide synthase 2* (*CerS2*)*, CerS4, CerS5, and CerS6; Sphingomyelin phosphodiesterase 1* (*Smpd1*), and *Smpd2; Ceramide kinase* (*Cerk*)*, Acyl-sphingosine amido-hydrolase-1(acid-Ceramidase)* (*Asah1*)*, Glucosylceramide synthase (Gcs).* Detailed information on primer sequences is listed in the Supplementary Table 1.

### Statistical analysis

All results were analyzed by using GraphPad Prism 8 software. Single statistical comparisons of a treatment group versus the control group were evaluated using Non-parametric *t*-test (Mann–Whitney test), whereas multiple group comparisons were evaluated using a one-way ANOVA test followed by a Tukey’s multiple comparison for adjustments. The *p < 0.05* values were considered significant.

## Results

### Acute TBI and activation of sphingomyelinases (SMases) in the brains

**Fig. 1:**
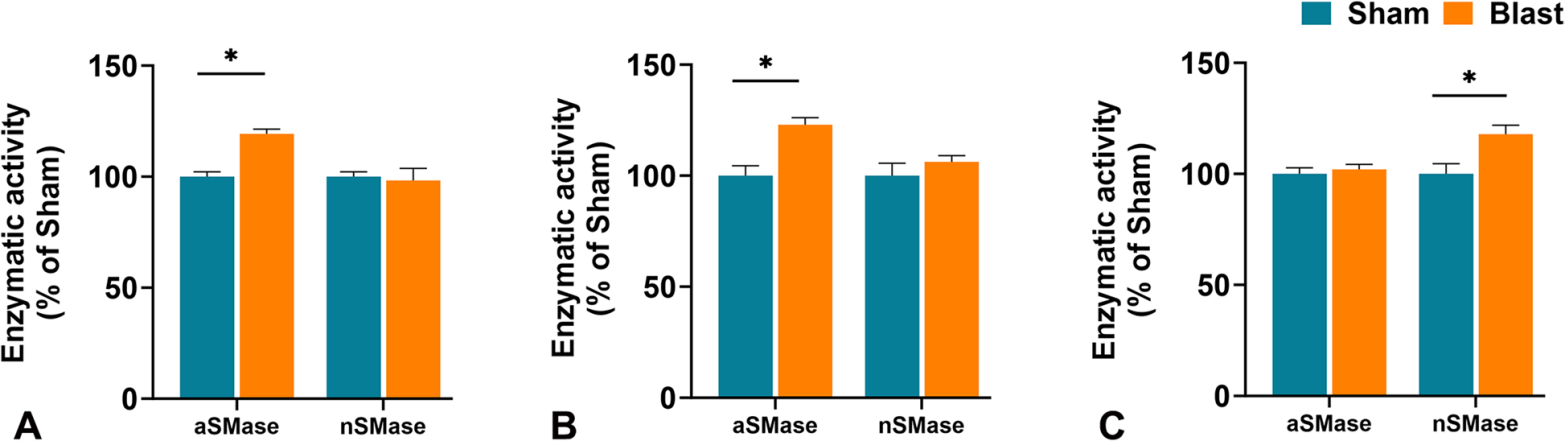
Analysis of enzymatic activity of sphingomyelinases (acidic sphingomyelinase: aSMase and basic sphingomyelinase: nSMase) in brain tissue of wild type (WT) mice at A) 1 day after treatment with 0-psi (sham) or 50-psi (blast) TBI, B) 3 days after treatment with 0-psi (sham) or 50-psi (blast) TBI, C) 7 days after treatment with 0-psi (sham) or 50-psi (blast) TBI. (*n =* 6; *p* < *0.05;* ANOVA)

The activation of sphingomyelinase enzymes (SMases) plays a crucial role in inducing inflammation [66]. This activation hydrolyzes sphingomyelin, releasing a cascade of bioactive lipids including ceramides, sphingosine, ceramide-1-phosphate and sphingosine-1-phosphate [66, 67]. Therefore*,* the study measured the enzymatic activity of sphingomyelinases, both acidic (aSMase) and neutral (nSMase), in the brain tissue of wildtype mice exposed to either Sham (Control) or Blast (TBI) and analyzed at 1, 3, and 7-days post-TBI. At 1 day post-TBI, Blast-exposed mice showed a significant elevation in aSMase activity compared to Sham mice; however, no changes were observed with nSMase activity (Fig. 1A). At 3 days post-TBI, a similar pattern was observed for both aSMase and nSMase, where aSMase activity increased significantly but not nSMase (Fig. 1B). However, at 7 days post-TBI, nSMase activity was found to be significantly higher in Blast-exposed brains, but not aSMase (Fig. 1C).

### Sphingolipid profile changes in the acute stages in post-TBI brains

**Fig. 2:**
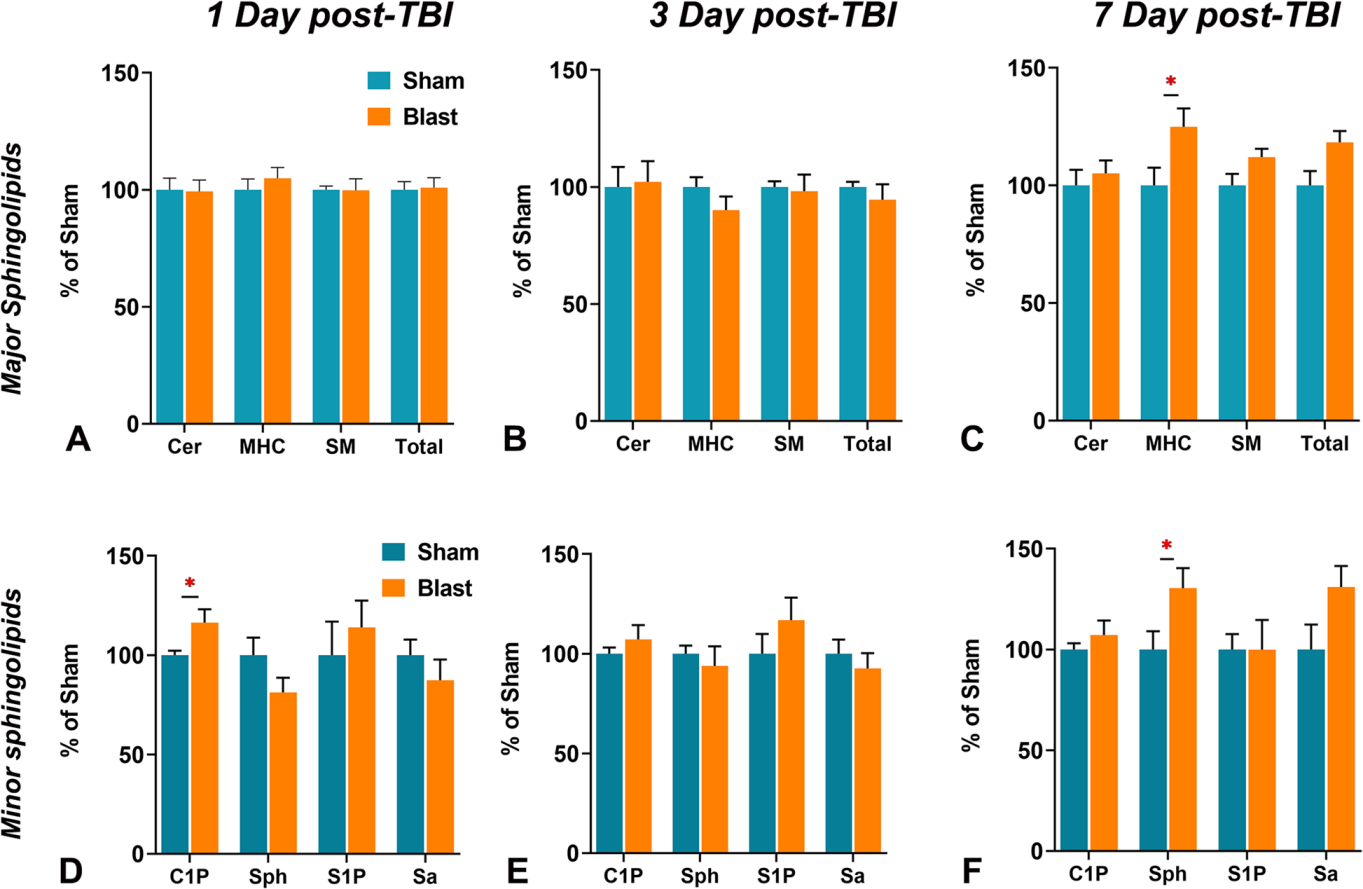
Analysis of different sphingolipids in the brain tissues of wild type (WT) mice 1 to 7 days after treatment with 0-psi (sham) or 50-psi (blast) TBI. **A** Ceramide (Cer), Monohexosylceramides (MHC), Sphingomyelins (SM), and total level of these sphingolipids (% of control WT-Sham) in the brain of WT-Sham (WT-0) and WT-Blast (WT-50) mice one day after TBI. **B** Ceramide-1-phosphate (C1P), Sphingosine (Sph), Sphingosine-1-phosphate (S1P), and Sphinganine (Sa) level (% of control WT-Sham) in the brain of four groups of animals as shown in A. (*n =* 6; *p* < *0.05*; ANOVA)

In an earlier study, our group reported significant alterations of ceramide levels in brain tissue of C57 mice one month after blast-induced TBI [52]. To identify mechanistic connections between sphingolipid changes and the development of TBI neuropathology, the present study focused on analyzing sphingolipids at the very acute stages of TBI. Using a targeted sphingolipids (SPLs) analysis by LC–MS/MS, the total tissue levels and composition of 12 major species of Ceramides C, monohexosylceramides (MHC), and sphingomyelins (SM) were determined. Levels of sphingosine (Sph), sphingosine-1-phosphate (S1P), and dihydrosphingosine or sphinganine (Sa) and ceramide-1-phosphate (C1P) were also measured. At 1 day post-TBI, there were no significant changes in total SPL as well in the major SPL classes, including total Cer, total MHC, and total SM (Fig. 2). However, an increase in total MHC levels was noticed at 7 days post-TBI (Fig. 2C). Additionally, there was an elevation in the levels of total ceramide-1-phosphate (C1P) at 1 day post-TBI (Fig. 2D) and sphingosine (Sph) at 7 days post-TBI (Fig. 2F).

### TBI-induced changes in individual SPL species in the brain

Although there were no major changes in the total levels of SPL and its major classes, such as Cer, MHC, and SM, compositional changes in those classes from an alteration in many individual species were found. At 1 day post-TBI, there were significantly elevated mol% levels of Cer C18:0 and SM C18:0 and decreased levels of Cer C22:0, Cer C24:1, MHC C22:0, and SM C26:1 in the Blast-exposed mice compared to the Sham-exposed mice brains (Table 1). After correction for multiple testing, the increase of Cer C18:0 and decrease in C24:1 remained highly significant (*p* < *0.0001 and p* < *0.001*, respectively) (Table 1). At 3 days post-TBI, there were significantly decreased mol% levels of Cer C14:0 and increased levels of MHC C20:0 in Blast-exposed brains compared to Sham (Table 1). After correction for multiple testing, the increase of MHC C20:0 remained significant (*p* < *0.05*) (Table 1). At 7 days post-TBI, there were significant changes in many SPL species that included increased levels of Cer C18:1, Cer C26:0; MHC C18:0, MHC C26:0; SM C24:0, SM C26:1, and SM C26:0 and decreased levels of Cer C22:0 in the Blast-exposed brains compared to Sham (Table 1). After correction for multiple testing, the increase of MHC (C18:0, C24:1) and SM (C18:0, C24:0) remained significant *p* < *0.05* for all) (Table 1).

**Table 1:** Brain mol% composition of Ceramide (Cer), Monohexosylceramide (MHC), and Sphingomyelin (SM) species from mice 1 day, 3 days, and 7 days after mild traumatic brain injury (mTBI) induced by 50-psi blast (Blast) or 0-psi (Sham) treatment

	***1 Day Post-TBI***				***3 Day Post-TBI***				***7 Day Post-TBI***			
***SPL Species***	**Sham**	**Blast**	***P*-value**	**Adjusted** ***P*-value**	**Sham**	**Blast**	***P*-value**	**Adjusted** ***P*-value**	**Sham**	**Blast**	***P*-value**	**Adjusted** ***P*-value**
Cer C14:0	0.36 ± 0.08	0.38 ± 0.07			0.36 ± 0.05	0.32 ± 0.01*	0.042	0.99	0.24 ± 0.02	0.24 ± 0.04		
Cer C16:0	2.57 ± 0.35	2.66 ± 0.36			2.74 ± 0.34	2.68 ± 0.40			2.98 ± 0.16	2.88 ± 0.38		
Cer C18:1	0.18 ± 0.03	0.17 ± 0.02			0.10 ± 0.07	0.15 ± 0.06			1.82 ± 0.17	2.54 ± 0.49*	0.005	
Cer C18:0	50.97 ± 2.45	56.12 ± 1.81*	0.003	< 0.0001	53.20 ± 2.07	52.46 ± 2.53			58.87 ± 1.93	58.31 ± 2.22		
Cer C20:0	5.99 ± 0.87	6.25 ± 1.12			5.55 ± 0.59	4.93 ± 0.58			4.27 ± 0.44	4.02 ± 0.84		
Cer C22:0	5.40 ± 0.61	4.54 ± 0.51*	0.022	0.612	4.13 ± 0.63	4.54 ± 0.44			4.49 ± 0.62	3.62 ± 0.45*	0.009	
Cer C24:1	26.77 ± 2.28	23.2 ± 1.88*	0.014	< 0.001	25.91 ± 2.52	26.84 ± 2.33			21.38 ± 0.93	22.03 ± 1.57		
Cer C24:0	7.40 ± 0.87	6.38 ± 1.24			7.53 ± 0.80	7.62 ± 0.66			5.23 ± 1.10	5.60 ± 0.62		
Cer C26:1	0.28 ± 0.08	0.21 ± 0.06			0.31 ± 0.06	0.27 ± 0.05			0.70 ± 0.07	0.72 ± 0.11		
Cer C26:0	0.08 ± 0.02	0.08 ± 0.04			0.11 ± 0.03	0.13 ± 0.09			0.04 ± 0.00	0.05 ± 0.01		
MHC C14:0	0.10 ± 0.02	0.09 ± 0.02			0.08 ± 0.02	0.08 ± 0.02			0.08 ± 0.02	0.10 ± 0.02		
MHC C16:0	1.99 ± 0.31	2.02 ± 0.36			2.37 ± 0.52	2.29 ± 0.65			1.30 ± 0.38	1.51 ± 0.22		
MHC C18:1	5.02 ± 0.55	5.18 ± 1.19			4.77 ± 0.95	4.05 ± 0.47			0.09 ± 0.01	0.10 ± 0.01		
MHC C18:0	7.87 ± 0.89	7.97 ± 1.16			8.33 ± 0.77	8.55 ± 1.14			7.93 ± 0.83	6.83 ± 0.17*	0.014	0.018
MHC C20:0	3.33 ± 0.23	3.41 ± 0.43			3.45 ± 0.35	4.67 ± 0.20*	0.0001	0.0074	3.14 ± 0.84	3.20 ± 0.28		
MHC C22:0	16.96 ± 0.50	16.07 ± 0.81*	0.034	0.33	16.15 ± 0.45	16.50 ± 0.73			16.11 ± 0.82	16.65 ± 0.71		
MHC C24:1	29.26 ± 1.78	29.98 ± 2.23			27.85 ± 0.64	28.15 ± 0.70			31.91 ± 1.38	32.50 ± 0.78*	0.08	0.0083
MHC C24:0	30.95 ± 0.36	30.75 ± 0.70			31.72 ± 1.33	30.76 ± 1.09			32.93 ± 1.13	32.97 ± 0.77		
MHC C26:1	3.26 ± 0.28	3.39 ± 0.48			3.6 ± 0.35	3.48 ± 0.38			5.00 ± 0.31	4.87 ± 0.46		
MHC C26:0	1.25 ± 0.25	1.16 ± 0.33			1.63 ± 0.36	1.40 ± 0.15			1.51 ± 0.33	1.27 ± 0.29*	0.005	0.58
SM C14:0	1.01 ± 0.15	0.91 ± 0.10			1.12 ± 0.17	1.12 ± 0.10			0.92 ± 0.15	1.09 ± 0.12		
SM C16:0	12.72 ± 0.27	12.75 ± 0.18			12.45 ± 0.28	12.23 ± 0.30			11.73 ± 0.30	11.70 ± 0.71		
SM C18:1	13.45 ± 0.15	13.63 ± 0.38			12.44 ± 0.31	12.14 ± 0.39			12.76 ± 0.68	12.39 ± 0.80		
SM C18:0	13.60 ± 0.25	14.14 ± 0.44*	0.023	0.11	12.70 ± 0.27	12.56 ± 0.51			12.31 ± 1.43	12.68 ± 0.34		0.018
SM C20:0	10.40 ± 0.35	10.25 ± 0.24			10.19 ± 0.36	9.98 ± 0.31			13.11 ± 0.40	13.04 ± 0.48*	0.046	
SM C22:0	8.07 ± 0.23	7.96 ± 0.41			9.27 ± 0.24	9.40 ± 0.66			10.90 ± 0.77	11.04 ± 0.41		
SM C24:1	21.58 ± 0.22	21.85 ± 0.39			20.69 ± 0.36	20.96 ± 0.64			21.61 ± 0.48	21.34 ± 0.50		
SM C24:0	17.41 ± 0.44	16.92 ± 0.65			18.48 ± 0.87	18.76 ± 0.93			15.09 ± 1.10	15.23 ± 0.76*	0.024	0.002
SM C26:1	1.44 ± 0.09	1.30 ± 0.09*	0.022	0.93	1.70 ± 0.25	1.84 ± 0.12			1.27 ± 0.23	1.20 ± 0.20*	0.028	0.90
SM C26:0	0.32 ± 0.02	0.29 ± 0.05			0.41 ± 0.04	0.42 ± 0.14			0.32 ± 0.03	0.30 ± 0.04*	0.003	0.99

Data presented as Mean ± SD (*n =* 5–7)

* Indicates significant changes (< 0.05). *P*-value determined by non-parametric *t*-test (Mann–Whitney test) and ‘Adjusted *P*-values’ by Tukey’s multiple comparison

At 1 day post-TBI, there was elevation in total ceramide-1-phosphate (C1P) (Fig. 2D). Among CIP species, measurable levels of C14:0, C16:0, C18:0, C22:0, C24:1, C24:0, and C26:0 were detected in mice brains. There was a significant increase in the levels of C1P and C18:0 (0.34 ± 0.04 vs. 0.22 ± 0.02 pmol/mg; p = 0.01) in the brains of Blast-exposed mice at 1-day post-TBI.

This study attempted to determine the temporal changes in the species of Cer, MHC, and SM over 1 day, 3 days, and 7 days post-TBI brains. The species that showed significant changes in their mole % either with time or between the groups (control vs. TBI) are shown in Supplemental Fig. S1. Most species showed concordance between control vs. TBI in their levels as a function of time but a small number showed differences in their mole % either at day 1 or day 7 (Cer C18:0, C18:1, C22:0, C24:1; MHC C18:0, C20:0, C22:0, C26:0; and SM C18:0 (Supplemental Fig. S1). The observed changes that include increases in the most abundant saturated Cer species C18:0 at 1 day post-TBI and very-long-chain Cer, MHC, and SM species at 7 days post-TBI may have implications with the TBI pathology.

### Expression of SPL metabolic marker genes in the brain tissue

**Fig. 3:**
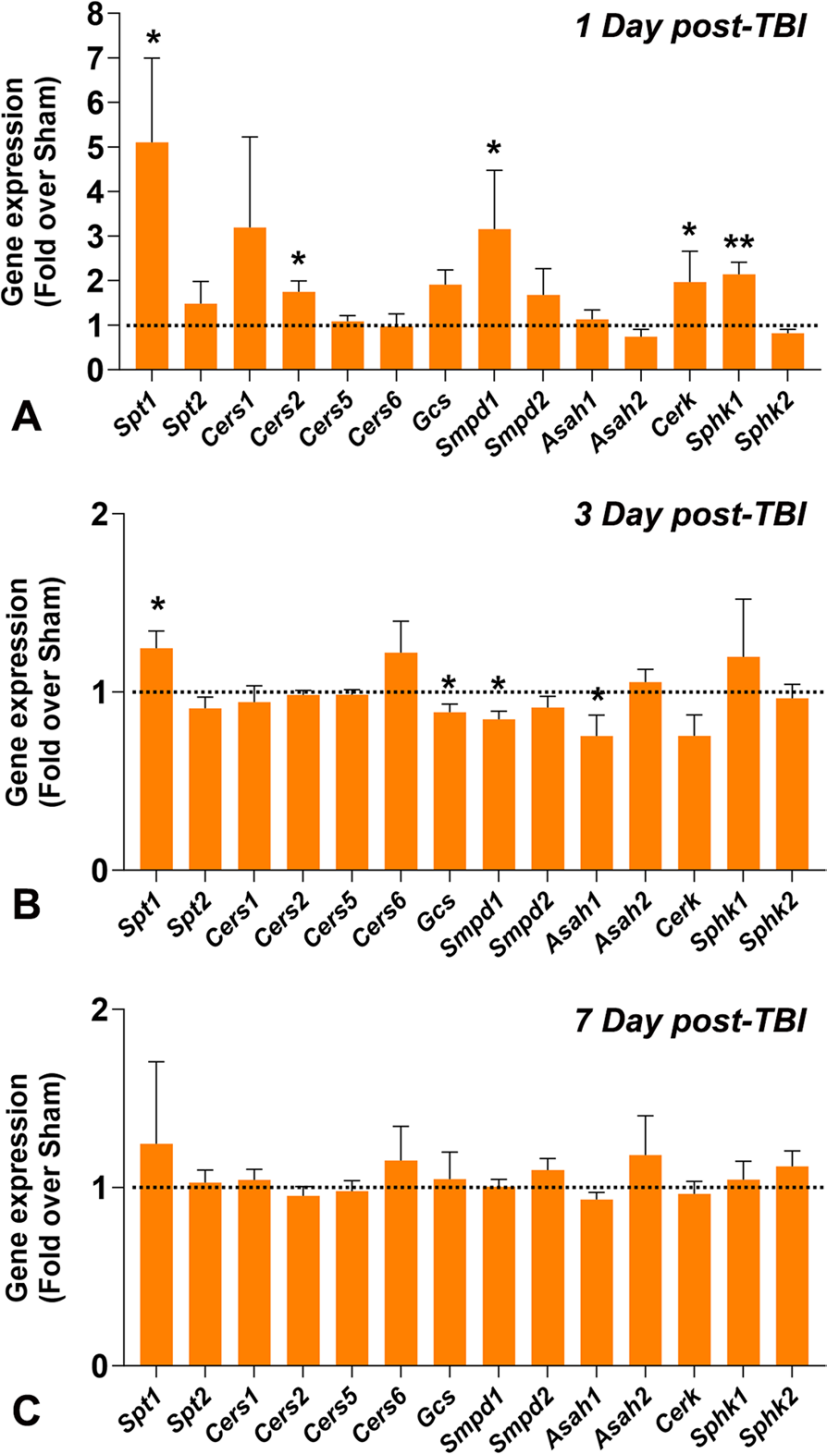
Expression analysis of sphingolipid metabolic genes in the brain tissues of wild type (WT) mice A) 1 day after treatment with 0-psi (sham) or 50-psi (blast) TBI, B) 3 days after treatment with 0-psi (sham) or 50-psi (blast) TBI, C) 7 days after treatment with 0-psi (sham) or 50-psi (blast) TBI. mRNA expression levels are presented over wild type sham (WT-0) (= 1.0) after normalization with two housekeeping genes, Ribosomal protein L19 (Rpl19) and Glyceraldehyde-3-phosphate dehydrogenase (Gapdh) (*n =* 6; *p* < *0.05*; ANOVA). Spt1, Serine palmitoyl transferase 1; Spt2; CerS1, Ceramide synthase 1; CerS2; CerS5; CerS6; GCS, Glucosyl-ceramide-synthase; Asah1, Acyl-sphingosine amido-hydrolase-1(acid-Ceramidase); Asah2; Smpd1, Sphingomyelin phosphodiesterase 1; Smpd2; Cerk, Ceramide kinase; Sphk1, Sphingosine kinase 1; Sphk2

qRT-PCR was used to quantify changes in gene expression of SPL metabolic markers in the brain tissue of mice exposed to Sham and Blast at 1, 3, and 7 days post-TBI. At 1 day post-TBI, out of 14 genes acquired, there was a significant elevation (fivefold) in the expression of de novo Cer biosynthetic gene, *Spt1 (Serine palmitoyltransferase 1*) in the Blast-exposed brains compared to Sham (Fig. 3A)*.* Moreover, there were significant increases in the expression of *Cers2* (*Ceramide synthase 2*)*, Cerk* (*Ceramide kinase*)*, Smpd1* (*Sphingomyelin phosphodiesterase 1*)*,* and *Sphk1* (*Sphingosine kinase 1*) in the Blast-exposed mice brains compared to Sham (Fig. 3A). At 3 days post-TBI, Blast-exposed mice showed a significant increase in *Spt1* but significant decreases in *Gcs* (*Glucosylceramide synthase*)*, Smpd1,* and *Asah1* (*Acylsphingosine amidohydrolase 1* or *acid-Ceramidase*) expression compared to Sham mice (Fig. 3B). At 7 days post-TBI, there were no differences in the expression of 14 SPL genes investigated between the Blast-exposed and Sham-exposed brains (Fig. 3C).

### Changes of SPL profile in the plasma

**Fig. 4:**
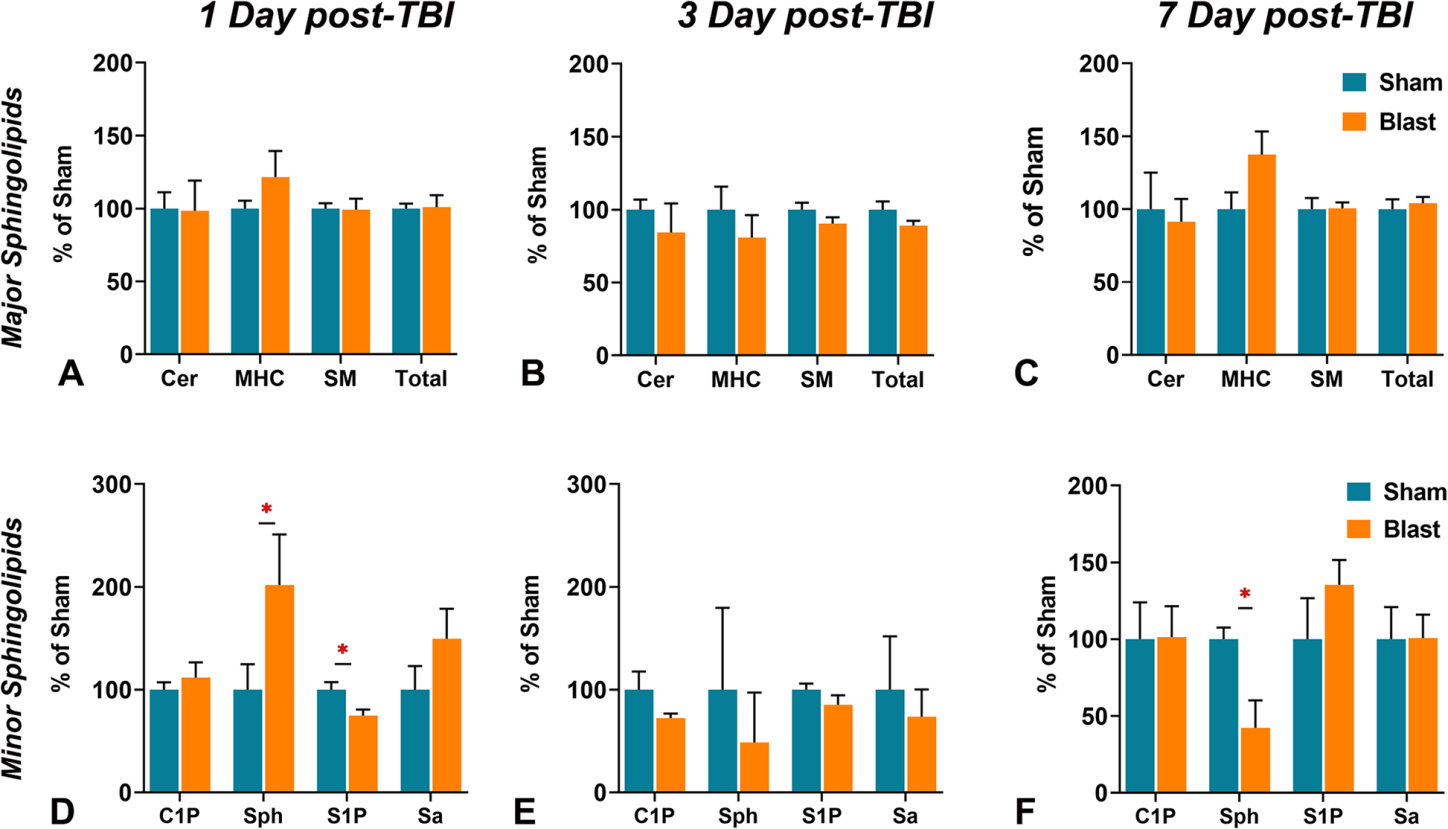
Analysis of different sphingolipids in plasma of wild type (WT) mice 1 to 7 days after treatment with 0-psi (sham) or 50-psi (blast) TBI. **A** Ceramide (Cer), Monohexosylceramides (MHC), Sphingomyelins (SM), and total level of these sphingolipids (% of control WT-Sham) in plasma of WT-Sham (WT-0) and WT-Blast (WT-50) mice one day after TBI. **B** Ceramide-1-phosphate (C1P), Sphingosine (Sph), Sphingosine-1-phosphate (S1P), and Sphinganine (Sa) level (% of control WT-Sham) in plasma of four groups of animals as shown in A. (*n =* 6; *p* < *0.05*; ANOVA)

Plasma SPL has been investigated as a biomarker for various neurodegeneration, neuroinflammation, and psychiatric diseases [37–44]. To determine if there is any effect of traumatic head injury on the plasma profile of sphingolipids, the profiles of SPL in the plasma from both Blast-exposed and Sham-exposed mice were analyzed at days 1, 3, and 7 post-TBI. At 1 day post-TBI, there were no significant changes in total SPL as well in the major SPL classes, including total Cer, total MHC, and total SM (Fig. 4A). However, there was a significant increase in the levels of sphingosine (Sph) and a significant decrease in sphingosine-1-phosphate (S1P) in the Blast-exposed mice compared to Sham-exposed mice (Fig. 4D). At 3 days post-TBI, there were no differences in the total SPL and various SPL classes as well as in the levels of bioactive C1P, Sph, S1P and Sa between the Blast- and Sham exposed mice (Fig. 4B, E). At 7 days post-TBI, like day 1 and day 3, no changes in the total levels of major sphingolipids in the plasma were detected; however, there was significantly decreased Sph levels in Blast-exposed mice compared to Sham-exposed mice (Fig. 4C, F). There were also.

### TBI-induced changes in individual SPL species in plasma

At the individual species level, considerable changes in the plasma SPL in the relative mol% composition of chain-length variants in all classes of SPL were detected. At 1 day post-TBI, the Blast-exposed mice showed significant increases in Cer C16:0, C18:1, C18:0, and MHC C16:0 compared to Sham (Table 2). After correction for multiple testing, the decrease of Cer C22:0 and MHC C22:0 and increase in MHC C16:0 remained significant (*p* < *0.05* for all) (Table 2). At 3 days post-TBI, Blast-exposed mice had elevated mol% levels of Cer C24:1, MHC C14:0, and SM C14:0 in their plasma but decreased relative levels of MHC C26:1, and SM C20:0 and C22:0 (Table 2). After correction for multiple testing, the increase of Cer C24:1 and decrease in Cer C24:0, MHC C22:0, and SM C22:0 remained highly significant (*p* < *0.001, p* < *0.05, p* < *0.05, and p* < *0.05*, respectively) (Table 2). At 7 days post-TBI, Blast-exposed mice had significantly elevated levels of Cer C22:0, MHC C22:0, and SM C22:0 and decreased levels of Cer C24:1 and MHC C14:0, C24:1, and C24:0 compared to Sham-exposed mice (Table 2). After correction for multiple testing, the increase of Cer C22:0, SM C22:0, MHC C22:0 and decrease in Cer C24:1, MHC 24:1, and MHC C24:0 remained significant (*p* < *0.05* for all) (Table 2).

**Table 2:** Plasma mol% composition of Ceramide (Cer), Monohexosylceramide (MHC), and Sphingomyelin (SM) species from mice 1 day, 3 days, and 7 days after mild traumatic brain injury (mTBI) induced by 50-psi blast (Blast) or 0-psi (Sham) treatment

	***1 Day Post-TBI***				***3 Day Post-TBI***				***7 Day Post-TBI***			
***SPL Species***	**Sham**	**Blast**	***P*-value**	**Adjusted** ***P*-value**	**Sham**	**Blast**	***P*-value**	**Adjusted** ***P*-value**	**Sham**	**Blast**	***P*-value**	**Adjusted** ***P*-value**
Cer C14:0	0.25 ± 0.10	0.28 ± 0.10			0.15 ± 0.06	0.23 ± 0.10			0.18 ± 0.05	0.17 ± 0.09		
Cer C16:0	1.49 ± 0.49	2.88 ± 1.07*	0.014	0.87	1.57 ± 1.12	1.08 ± 0.48			4.40 ± 2.15	3.51 ± 1.65		
Cer C18:1	0.04 ± 0.02	0.09 ± 0.04*	0.015	0.99	0.06 ± 0.04	0.02 ± 0.01			0.39 ± 0.17	0.34 ± 0.15		
Cer C18:0	0.85 ± 0.36	1.26 ± 0.29*	0.04	0.99	0.61 ± 0.55	0.90 ± 0.56			3.19 ± 1.38	2.15 ± 1.39		
Cer C20:0	3.55 ± 0.87	3.47 ± 0.82			2.31 ± 1.27	2.30 ± 0.66			2.87 ± 0.97	2.78 ± 1.00		
Cer C22:0	24.39 ± 6.40	19.82 ± 3.14*	0.09	0.013	28.33 ± 4.68	25.23 ± 7.37			14.70 ± 1.08	19.51 ± 3.40*	0.012	0.0027
Cer C24:1	22.80 ± 4.99	26.15 ± 5.80			19.73 ± 5.80	29.94 ± 1.63*	0.006	< 0.0001	32.32 ± 2.17	28.13 ± 3.81*	0.04	0.012
Cer C24:0	44.75 ± 4.61	43.78 ± 6.75			46.04 ± 7.05	38.97 ± 5.45*	0.072	0.003	36.79 ± 3.52	39.11 ± 3.61		
Cer C26:1	0.37 ± 0.13	0.42 ± 0.05			0.18 ± 0.02	0.23 ± 0.08			3.93 ± 0.13	3.49 ± 0.68		
Cer C26:0	1.26 ± 0.88	1.46 ± 0.45			0.78 ± 0.41	0.95 ± 0.51			1.23 ± 0.55	0.83 ± 0.45		
MHC C14:0	0.16 ± 0.08	0.17 ± 0.04			0.07 ± 0.04	0.17 ± 0.08*	0.02	0.99	0.10 ± 0.03	0.07 ± 0.02*	0.043	0.99
MHC C16:0	17.14 ± 2.89	24.45 ± 5.53*	0.015	0.0003	15.57 ± 3.17	16.84 ± 4.04			15.42 ± 2.46	18.55 ± 3.42		
MHC C18:1	0.43 ± 0.13	0.54 ± 0.07			0.34 ± 0.08	0.31 ± 0.11			0.72 ± 0.14	0.71 ± 0.05		
MHC C18:0	1.15 ± 0.55	1.68 ± 0.54			0.64 ± 0.43	1.06 ± 0.61			3.20 ± 1.69	3.47 ± 0.91		
MHC C20:0	1.42 ± 0.09	1.38 ± 0.19			1.46 ± 0.69	1.23 ± 0.41			2.35 ± 0.21	2.62 ± 0.96		
MHC C22:0	30.32 ± 8,96	24.26 ± 4.01*	0.10	0.004	37.07 ± 6.17	30.11 ± 4.92*	0.05	0.009	22.95 ± 2.15	27.16 ± 1.87*	0.004	0.005
MHC C24:1	27.28 ± 4.55	26.94 ± 4.33			23.14 ± 4.74	27.91 ± 3.08			33.04 ± 2.96	28.90 ± 1.29*	0.005	0.006
MHC C24:0	21.45 ± 2.18	19.91 ± 3.28			21.09 ± 4.58	21.97 ± 5.14			21.11 ± 1.64	17.77 ± 3.10*	0.04	0.04
MHC C26:1	0.20 ± 0.11	0.27 ± 0.05			0.28 ± 0.13	0.08 ± 0.05*	0.011	0.99	0.62 ± 0.27	0.38 ± 0.28		
MHC C26:0	0.40 ± 0.09	0.35 ± 0.16			0.29 ± 0.08	0.28 ± 0.15			0.49 ± 0.16	0.37 ± 0.14		
SM C14:0	1.88 ± 0.47	1.67 ± 0.16			1.42 ± 0.39	2.04 ± 0.30*	0.02	0.92	1.35 ± 0.11	1.31 ± 0.30		
SM C16:0	19.94 ± 2.02	21.81 ± 3.04			20.19 ± 1.27	20.23 ± 0.51			20.32 ± 2.54	19.97 ± 0.73		
SM C18:1	1.85 ± 0.69	2.02 ± 0.46			1.27 ± 0.30	1.61 ± 0.65			2.62 ± 0.81	2.21 ± 0.68		
SM C18:0	5.09 ± 1.78	6.52 ± 1.23			3.77 ± 1.25	5.01 ± 1.91			7.03 ± 1.26	6.37 ± 2.52		
SM C20:0	3.75 ± 0.63	3.72 ± 0.70			4.42 ± 0.41	3.95 ± 0.31*	0.049	0.96	4.37 ± 1.00	4.50 ± 0.77		
SM C22:0	15.72 ± 3.84	13.10 ± 1.35			19.23 ± 2.67	15.81 ± 2.39*	0.043	0.004	14.45 ± 1.76	16.79 ± 1.68*	0.03	0.03
SM C24:1	30.29 ± 1.33	30.90 ± 1.47			28.30 ± 1.87	29.92 ± 1.57			30.22 ± 1.88	29.63 ± 0.86		
SM C24:0	20.15 ± 2.05	18.70 ± 3.26			20.13 ± 2.49	20.32 ± 1.35			18.74 ± 2.63	18.66 ± 2.22		
SM C26:1	0.31 ± 0.12	0.32 ± 0.10			0.23 ± 0.08	0.29 ± 0.09			0.46 ± 0.24	0.30 ± 0.10		
SM C26:0	0.28 ± 0.18	0.31 ± 0.17			0.22 ± 0.17	0.27 ± 0.11			0.45 ± 0.41	0.25 ± 0.13		

Data presented as Mean ± SD (*n =* 5–7)

* Indicates significant changes (< 0.05). *P*-value determined by non-parametric *t*-test (Mann–Whitney test) and ‘Adjusted *P*-values’ by Tukey’s multiple comparison

This study also analyzed the temporal changes in the species of Cer, MHC, and SM over 1 day, 3 days, and 7 days post-TBI plasma. The species that showed significant changes in their mole % either with time or between the groups (control vs. TBI) are shown in Supplemental Fig. S2. The SPL species that showed discordance between control vs. TBI in their levels as a function of time include Cer C24:0; MHC C14:0, C16:0, C22:0, C24:0, C24:1, C26:1; and SM C14:0, C20:0 and C22:0 (Supplemental Fig. S2).

### Comparison of ratios of sphingolipid species

Historically, specific ratios SPL species in the plasma have been documented as biomarkers for systemic or neurodegenerative diseases [68–70]. This study analyzed the ratios of individual species in all classes of SPL in various combinations and examined if they were different between Blast-exposed mice and Sham mice. At 1 day post-TBI, after multiple comparison adjustments in *P-values*, plasma ratios of Cer C24:1/C18:1 and MHC C24:1/C26:1 were significantly reduced in the Blast-exposed mice compared to the Sham-exposed plasma (Table 3). Very interestingly, a complete reversal (significantly increased) of those two ratios of Cer and MHC occurred at 3 days post-TBI plasma, suggesting their association with the exposure of TBI (Table 3). At 7 days post-TBI, there was a decrease in the plasma ratio of MHC C24:0/C22:0 in the Blast-exposed mice compared to the Sham-exposed plasma (Table 3).

**Table 3:** Comparison of ratios in various combinations of plasma sphingolipid species in mice with or without Blast exposure at 1 Day, 3 Day, and 7 Day post-Blast

SPL Ratios	*1 Day post-TBI*		*3 Day post-TBI*		*7 Day post-TBI*	
	Sham	Blast	Sham	Blast	Sham	Blast
**Cer**						
18:0/16:0	0.56 ± 0.1	0.46 ± 0.1	0.39 ± 0.1	0.79 ± 0.5	0.75 ± 0.2	0.55 ± 0.2
18:0/20:0	0.25 ± 0.1	0.37 ± 0.1	0.24 ± 0.1	0.37 ± 0.2	1.15 ± 0.6	0.69 ± 0.3
18:0/22:0	0.04 ± 0.0	0.07 ± 0.0	0.02 ± 0.0	0.04 ± 0.0	0.22 ± 0.1	0.12 ± 0.1
18:0/24:0	0.02 ± 0.0	0.03 ± 0.0	0.01 ± 0.0	0.02 ± 0.0	0.09 ± 0.0	0.06 ± 0.0
18:0/18:1	27.39 ± 13.3	16.91 ± 7.6	19.27 ± 22.4	37.12 ± 10.6	8.21 ± 3.0	5.76 ± 2.5
24:0/24:1	2.05 ± 0.5	1.77 ± 0.6	2.62 ± 1.3	1.31 ± 0.2	1.14 ± 0.1	1.42 ± 0.3
26:0/26:1	3.21 ± 1.3	3.51 ± 1.0	4.24 ± 2.2	3.90 ± 1.4	0.31 ± 0.1	0.25 ± 0.1
22:0/20:0	7.40 ± 3.5	5.86 ± 1.2	14.88 ± 5.9	12.88 ± 9.3	5.67 ± 2.2	8.45 ± 4.8
24:0/22:0	1.93 ± 0.5	2.29 ± 0.7	1.67 ± 0.4	1.67 ± 0.2	2.50 ± 0.2	2.05 ± 0.4
26:0/24:0	0.03 ± 0.0	0.03 ± 0.0	0.02 ± 0.0	0.02 ± 0.0	0.03 ± 0.0	0.02 ± 0.0
24:1/18:1	802.7 ± 359	409.4 ± 317.5*	612.8 ± 663.4	2121.5 ± 1953.3***	96.5 ± 44.8	109.3 ± 71.4
24:1/26:1	66.59 ± 24.3	63.72 ± 17.5	105.80 ± 23.2	153.1 ± 86.4	8.23 ± 0.7	8.33 ± 1.9
**MHC**						
*18:0/16:0*	0.06 ± 0.0	0.07 ± 0.0	0.04 ± 0.0	0.06 ± 0.0	0.22 ± 0.1	0.20 ± 0.1
*18:0/20:0*	0.81 ± 0.4	1.22 ± 0.4	0.43 ± 0.1	0.84 ± 0.3	1.32 ± 0.6	1.58 ± 0.8
*18:0/22:0*	0.04 ± 0.0	0.07 ± 0.0	0.02 ± 0.0	0.04 ± 0.0	0.14 ± 0.1	0.13 ± 0.0
*18:0/24:0*	0.05 ± 0.0	0.09 ± 0.0	0.03 ± 0.0	0.05 ± 0.0	0.16 ± 0.1	0.20 ± 0.1
*18:0/18:1*	2.56 ± 0.7	3.07 ± 0.7	1.78 ± 0.8	3.51 ± 1.8	4.73 ± 2.6	4.98 ± 1.5
*24:0/24:1*	0.80 ± 0.1	0.74 ± 0.1	0.99 ± 0.5	0.81 ± 0.3	0.65 ± 0.1	0.62 ± 0.1
*26:0/26:1*	2.65 ± 1.8	1.32 ± 0.7	1.34 ± 1.0	3.90 ± 2.0	1.00 ± 0.6	1.30 ± 0.7
*22:0/20:0*	21.32 ± 6.2	17.61 ± 1.5	29.77 ± 11.9	28.57 ± 17.0	9.80 ± 1.1	12.26 ± 6.2
*24:0/22:0*	0.77 ± 0.3	0.84 ± 0.2	0.58 ± 0.2	0.76 ± 0.3	0.92 ± 0.1	0.66 ± 0.1*
*26:0/24:0*	0.02 ± 0.0	0.02 ± 0.0	0.01 ± 0.0	0.01 ± 0.0	0.02 ± 0.0	0.02 ± 0.0
*24:1/18:1*	65.82 ± 12.5	50.80 ± 12.0	68.05 ± 10.2	104.4 ± 51.9	47.09 ± 7.7	41.05 ± 3.4
*24:1/26:1*	186.9 ± 140	101.7 ± 11.5*	100.3 ± 58.9	443.0 ± 223.8***	61.9 ± 26.5	97.7 ± 35.8
**SM**						
18:0/16:0	0.25 ± 0.1	0.30 ± 0.1	0.19 ± 0.1	0.25 ± 0.1	0.35 ± 0.0	0.32 ± 0.1
18:0/20:0	1.45 ± 0.7	1.84 ± 0.7	0.86 ± 0.3	1.29 ± 0.6	1.71 ± 0.7	1.38 ± 0.4
18:0/22:0	0.36 ± 0.2	0.51 ± 0.2	0.21 ± 0.1	0.33 ± 0.2	0.50 ± 0.2	0.39 ± 0.2
18:0/24:0	0.26 ± 0.1	0.36 ± 0.1	0.19 ± 0.1	0.25 ± 0.1	0.39 ± 0.1	0.36 ± 0.2
18:0/18:1	2.78 ± 0.1	3.25 ± 0.3	2.94 ± 0.4	3.13 ± 0.1	2.81 ± 0.7	2.80 ± 0.4
24:0/24:1	0.67 ± 0.1	0.61 ± 0.1	0.72 ± 0.1	0.68 ± 0.1	0.63 ± 0.1	0.63 ± 0.1
26:0/26:1	0.84 ± 0.2	0.92 ± 0.3	0.87 ± 0.4	0.94 ± 0.1	0.84 ± 0.4	0.84 ± 0.2
22:0/20:0	4.16 ± 0.3	3.59 ± 0.5	4.36 ± 0.6	4.03 ± 0.7	3.37 ± 0.2	3.87 ± 1.0
24:0/22:0	1.32 ± 0.2	1.42 ± 0.2	1.07 ± 0.2	1.30 ± 0.2	1.30 ± 0.1	1.11 ± 0.1
26:0/24:0	0.01 ± 0.0	0.02 ± 0.0	0.01 ± 0.0	0.01 ± 0.0	0.02 ± 0.0	0.01 ± 0.0
24:1/18:1	18.32 ± 6.6	15.81 ± 3.0	23.33 ± 6.2	21.28 ± 11.6	12.17 ± 2.7	14.92 ± 5.7
24:1/26:1	105.8 ± 29.6	105.9 ± 38.5	139.9 ± 69.4	113.5 ± 49.3	80.9 ± 41.8	111.2 ± 40.1

Data presented as Mean ± SD analyzed by Two-way ANOVA, and the *P*-values were determined between the comparison groups by Tukey’s multiple comparisons

*p < 0.05, ***p < 0.001 between Blast (*n =* 6) and Sham-treated mice (*n =* 6)

A comparison of changes among the SPL species in plasma and brain tissues following mTBI is presented in Supplemental Table 1. The changes in the plasma profile of SPL species, some of their particular ratios, and their temporal variability indicate their association with exposure of the brain to TBI and may serve as biomarkers for the prognosis of traumatic head injury.

## Discussion

Associations of sphingolipid signaling with neuroinflammation pathology have been well documented and predicted to be a promising target for therapeutic management as well as a potential prognostic marker in neurodegenerative diseases [71, 72]. In a similar pathological context, TBI involves neuroinflammation-mediated neurodegeneration where aberrations in sphingolipid metabolism have been demonstrated [49, 73]. Our previous study with a mouse model of mild TBI (mTBI) revealed changes in brain SPL profiles even after 30 days post-TBI [52]. In this study, brain and plasma SPL profile changes were observed within the first seven days following exposure to mTBI, which could be associated with the early pathophysiology of the TBI process.

The major findings in the brain tissue included significant elevation of 1) acid sphingomyelinase (aSMase) at 1 and 3 days post-TBI but no changes in nSMase levels (Fig. 1); 2) elevation of nSmase levels at 7 days post-TBI (Fig. 1); 3) C1P levels at 1 day post-TBI (Fig. 2); 4) monohexosylceramide (MHC) and sphingosine at 7 days post-TBI (Fig. 2); 5) relative mole% of Cer C18:0 at 1 day (Table 1), MHC C20:0 at 3 days (Table 1), and MHC (C18:0, C24:1) and sphingomyelins (SM) (C18:0, C24:0) at 7 days **(**Table 1); and 6) significant increase of several SPL metabolic genes at 1-day, reduced numbers at 3-days and none at 7-days post-TBI (Fig. 3). The major findings in plasma included 1) a significant increase in MHC C16:0 as well as a decrease in C22:0 in Cer and MHC classes at 1 day (Table 2); 2) a significant increase in long-chain Cer (C24:1) accompanied by decreases in Cer C24:0 and C22:0 in MHC and SM classes at 3 days (Table 2); 3) a significant increase of C22:0 in all classes of SPL (Cer, MHC and SM) as well as a decrease in Cer C24:1, MHC C24:1 and MHC C24:0 at day 7 (Table 2); and 4) a significant elevation of Sph and reduction of S1P at 1 day; and 5) a significant reduction of Sph at day 7 (Fig. 4).

This study also explored the relationship between SPL metabolites in plasma and brain tissues following mTBI (Supplemental Table 1). Most significant metabolites demonstrated an inverse relationship between brain and plasma. Notably, Cer C24:1, MHC C20:0, and MHC C24:1 exhibited significant alterations in both brain and plasma. Interestingly, there was a significant elevation of MHC C24:1 in plasma and reduction in brain at 7 days post-TBI. This observed inverse relationship may be attributable to the complex interplay of central and systemic inflammatory responses following a mTBI. After a TBI, chemokines and other inflammatory molecules can leak through a compromised blood–brain barrier into the systemic circulation, attracting cells of the peripheral immune system to the site of injury and potentially activating an overactive inflammatory response known as systemic immune response syndrome [74]. Thus, negative feedback to systemic inflammation is often provided by the hypothalamus–pituitary–adrenal axis [74]. This adaptive response may aim to mitigate inflammatory damage, highlighting a sophisticated interplay between central and peripheral immune responses post-TBI.

In order to design therapeutic interventions for TBI, it is imperative to understand the pathophysiology of TBI by designing animal models. The procedure used in this study is an over-pressure air blast using a device developed and standardized by our group to induce TBI in mice using a small horizontally mounted air cannon system [17, 51–53, 75, 76]. This model simulates a closed-head primary blast injury with forces similar to those seen in an explosion or blunt force-mediated human mild-TBI (mTBI). Over the past decade, our research team has performed an extensive neurological and behavioral characterization of the mice exposed to this TBI procedure. This TBI caused by a single 50–60 psi blast in mice results in diffuse axonal injury in white matter tracts, characterized by swollen axonal bulbs, during the first few days [51, 75–78], followed by degeneration of injured axons. These mice exhibit clear motor deficiency in the early phase of recovery (1–2 weeks) and demonstrate persistent microglial activation in the major fiber tracts and degeneration of neurons in various brain regions at 2–3 months post-TBI. These pathologic characteristics are consistent with human mTBI; therefore, this procedure can act as a model for mTBI in mice.

Although several mechanisms in TBI have been proposed, a growing consensus favors the critical role of brain lipids and lipid pathway enzymes, including SMase activation and glia-mediated neuroinflammation [16, 79]. Activation and signaling of the enzymes involved in the metabolism of SPLs, especially that of bioactive SPLs, such as neutral and acid sphingomyelinase (nSMase, aSMase), ceramidase, and sphingosine kinase (SphK), have been linked to the development of various inflammatory and neurological diseases [80–84]. SMase hydrolyzes SM to produce phosphocholine, and Cer is a key step in SPL signaling. Like in many other neurodegenerative conditions, activation of SMases and Cer-induced neuroinflammatory pathology have been documented in TBI [85–88]. This study indicates activation of aSMase in the acute damage phase but nSMase in the chronic phase (Fig. 1). Our previous study also supports this notion of no changes in aSMase at 30-day post-TBI, but nSMase increased significantly [52]. This suggests that TBI’s initial insult impacts or is associated with aSMase activity, whereas chronic inflammatory effects are associated with nSMase activity. Prior studies have reported that aSMase-Cer axis regulates the pathogenesis of Major Depressive Disorder (MDD) which can occur after TBI [89]. Niziloket al., observed a similar pathology in relation to aSMase-Cer axis and accumulation of phosphorylated-tau protein in the mouse model of TBI [87]. Moreover, inhibition of aSMase activity by a single injection of amitriptyline, an inhibitor of aSMase, 1 h after TBI decreased accumulation of p-tau in the hippocampus one month after injury and decreased levels of depression [87]. Genetically ablated *Smpd1*^*−/−*^ (aSMase) also reproduced similar results, indicating a major role of aSMase activation in the initiation of TBI neuropathology [87]. In another study modeling TBI in mice, inhibiting aSMase by intraperitoneal imipramine significantly reduced levels of Cer and aSMase activity in the brain tissue, neuronal death, and cognitive dysfunction [90]. In the context of these prior studies, these findings align well with other experimental models of Cer-regulated neuroinflammation-mediated neurodegeneration in TBI and suggests aSMase could be a potential therapeutic target in the acute phases of TBI, whereas nSMase for chronic phases of TBI.

Another important finding was an elevation of ceramide-1-phosphate (C1P) in the brains of blast-exposed mice at 1-day post-TBI (Fig. 2). Phosphorylation of Cer by ceramide kinase (CK) generates C1P, a process known to activate cytosolic phospholipase A_2_ (cPLA_2_), which is crucial in producing proinflammatory eicosanoids from arachidonic acid (AA) by COX and LOX enzymes [66, 91–93]. The data suggests potential roles of C1P in generating proinflammatory lipids (eicosanoids) and acute inflammation in TBI.

This study also found significantly elevated level of Cer C18:0 in the blast-exposed brain at 1 day post-TBI (Table 1). Pathological elevation of Cer C18:0 has been observed in the cerebrospinal fluid of stroke patients [94]. In ischemic brain injury in mice, Cer C16:0 and Cer C18:0 have been found to be increased at 3 h post-injury, and de novo synthesis of Cer has been proposed [95]. Moreover, a study by Nielson et al. reported an increased level of Cer C18:1 in their model on focal cerebral ischemia at 5-days of ischemic injury and simultaneous elevation of C1P and S1P 7 days post-injury [96]. The elevation of Cer and Cer-mediated upregulation of proinflammatory factors are well known for inducing apoptosis, parthanatos, and neurodegeneration [97, 98]. Cer C18:0 can be converted to CIP by ceramide kinases, and the present study found that ceramide-1-phosphate (C1P) was elevated in the brains of blast-exposed mice at 1-day post-TBI (Fig. 2).

Elevation of Cer precursor dihydrosphingosine (Sa) at 1, 2 and 7 days post-injury in animal models suggests de novo synthesis of Cer [99]; however, the present study did not find significantly different levels of Sa between Blast-exposed and Sham-exposed mice. In this study, there were no significant alterations in the total levels of Cer detected; however, there was a noteworthy increase in the total MHC species at 7 days post TBI in Blast-induced brains compared to the sham condition (Fig. 2C). To maintain cellular homeostasis, elevated Cer get quickly converted to MHC to reduce its adverse effects, which may explain this study’s findings of increasing MHC levels in the brains of 7 days post-TBI [100]. Cer can also be generated from SMase activation as well as from de novo synthesis [101]. Significant elevation of de novo pathway genes (*Spt1*) at both 1 and 3 days, CerS2 at 1 day may indicate activation of de novo pathway and at the same time elevation of Smpd1 may associate with aSMase activation, Cerk with C1P generation, and Sphk1 for S1P (Fig. 3).

Plasma sphingolipids have been increasingly recognized as potential biomarkers for neurodegenerative diseases, which prompted this investigation into their role in TBI. In animal models of stroke, an elevation of plasma long-chain Cer and long-chain SM has been demonstrated at 24 and 48 h post-injury, respectively [102]. In this study’s TBI model, plasma long-chain Cer C24:1 increased significantly after three days post-TBI (Table 2). In Parkinson’s disease, higher plasma Cer C16, C18, C20, C22, and C24:1 have been associated with worse cognition [103]. Similar changes in plasma Cer have also been reported in dementia, with increasing level of C18:1 and C24:1 MHC predicting severe pathology in Alzheimer’s Disease [104]. These pathological alterations of plasma SPL metabolites in neurodegenerative disorders and stroke suggest its potential role as a TBI biomarker in predicting outcomes of patients and TBI severity. To search for the potential metabolites in association with TBI severity, one study group recruited a cohort of 716 patients with TBI and compared it with non-TBI reference patients. They observed a significant increase in Cer levels but a significant decrease in SM levels between groups [105]. Moreover, increasing severity of TBI was associated with a decrease in SM, which was supported by clinicopathological parameters such as head CT [105]. With an abundance of research suggesting a strong association of lipid markers with the severity of TBI patients, lipids show promise as potential biomarkers for predicting TBI severity and merit further investigation for their clinical applicability.

Recently, US Food and Drug Administration (FDA) has approved a combination of two disease-specific biomarkers, one is the ubiquitin C-terminal hydrolase-L1 (UCH-L1) for neurons, and the other one is a glial fibrillary acidic protein (GFAP) for astrocytes for effective diagnosis of mild TBI having a brain lesion [106–108]. Although protein biomarkers effectively measure tissue damage, they often lack disease specificity and do not provide insights into metabolic disruption, one of the most significant events in TBI-associated energy failure/crisis [109]. As lipids are one of the major constituents of the brain and have the ability to cross the blood–brain barrier, evaluating lipid metabolites is a strength in this study and could be one of the best approaches in biomarker identification that addresses metabolic disruption. TBI pathology is a protracted form of neuroinflammation-mediated neurodegeneration, and lipids could act as either pan-inflammatory markers (ceramide, arachidonic acid), pan-anti-inflammatory markers (eicosapentaenoic acid and docosahexaenoic acid), and other bioactive intermediates (eicosanoids, prostaglandins). As with all studies, it is important to consider this study within the context of its limitations. Limitations of the TBI model exist as it negates certain variables, such as hypoxia from blast-induced pulmonary injury, which may independently contribute to neuronal stress and damage [17, 52, 53, 75, 76]. Additionally, this study ensured meticulous pre-analytical stability of samples with a standardized protocol, and the results demonstrate consistent levels of sphingolipids and their enzymes across the sample groups, indicating uniformity in sample handling and processing. However, despite these precautions, subtle variations in pre-analytical procedures could still potentially impact the results. Moreover, analysis was limited by a small sample size, preventing advanced statistical techniques such as ternary plots or logistic regression [110]. It is important to validate these findings and undertake more comprehensive analyses with larger sample sizes.

## Conclusions

The present study aimed to elucidate the status of inflammatory SPL metabolites in the acute stage of TBI and explore the potential of SPL metabolites as biomarkers for TBI. In this study, TBI was induced under controlled conditions, and further research in other TBI animal models and human subjects is necessary to assess the effect of the types of TBI and their severity on the brain and circulating lipids to understand their pathophysiological connections. In conclusion, SPL metabolites in the plasma hold immense promise as biomarkers in TBI, and harnessing their capabilities may prove useful in clinical settings. The goal is to give providers the ability to objectively assess the severity and progression of TBI one day, allowing for a more accurate diagnosis, prognosis, and treatment monitoring. It is imperative to continue conducting more studies to determine the usefulness and accuracy of SPL biomarkers in TBI.

### Supplementary Information

Supplementary Material 1: Supplemental Table 1. Comparative Analysis of Significant Metabolite Changes in Brain and Plasma Following Traumatic Brain Injury (TBI). Supplemental Figure S1. Longitudinal Variability of Brain Sphingolipid Species: Temporal changes in the levels of Ceramide (Cer), Monohexosylceramide (MHC), and Sphingomyelin (SM) species. Supplemental Figure S2. Longitudinal Variability of Plasma Sphingolipid Species: Temporal changes in the levels of Ceramide (Cer), Monohexosylceramide (MHC), and Sphingomyelin (SM) species.

## Data Availability

All materials and data will be available from the corresponding author following University of Tennessee’s policy of sharing research materials and data.
