# Supplementary material for: Sphingolipid changes in mouse brain and plasma after mild traumatic brain injury at the acute phases
Source: Lipids Health Dis. 2024 Jun 27;23:200. doi: 10.1186/s12944-024-02186-x (PMC11209960; doi:10.1186/s12944-024-02186-x)
Supplement: Supplementary file 1 — Supplementary Material 1: Supplemental Table 1. Comparative Analysis of Significant Metabolite Changes in Brain and Plasma Following Traumatic Brain Injury (TBI). Supplemental Figure S1. Longitudinal Variability of Brain Sphingolipid Species: Temporal changes in the levels of Ceramide (Cer), Monohexosylceramide (MHC), and Sphingomyelin (SM) species. Supplemental Figure S2. Longitudinal Variability of Plasma Sphingolipid Species: Temporal changes in the levels of Ceramide (Cer), Monohexosylceramide (MHC), and Sphingomyelin (SM) species. [file 12944_2024_2186_MOESM1_ESM.pdf]

Figure S1

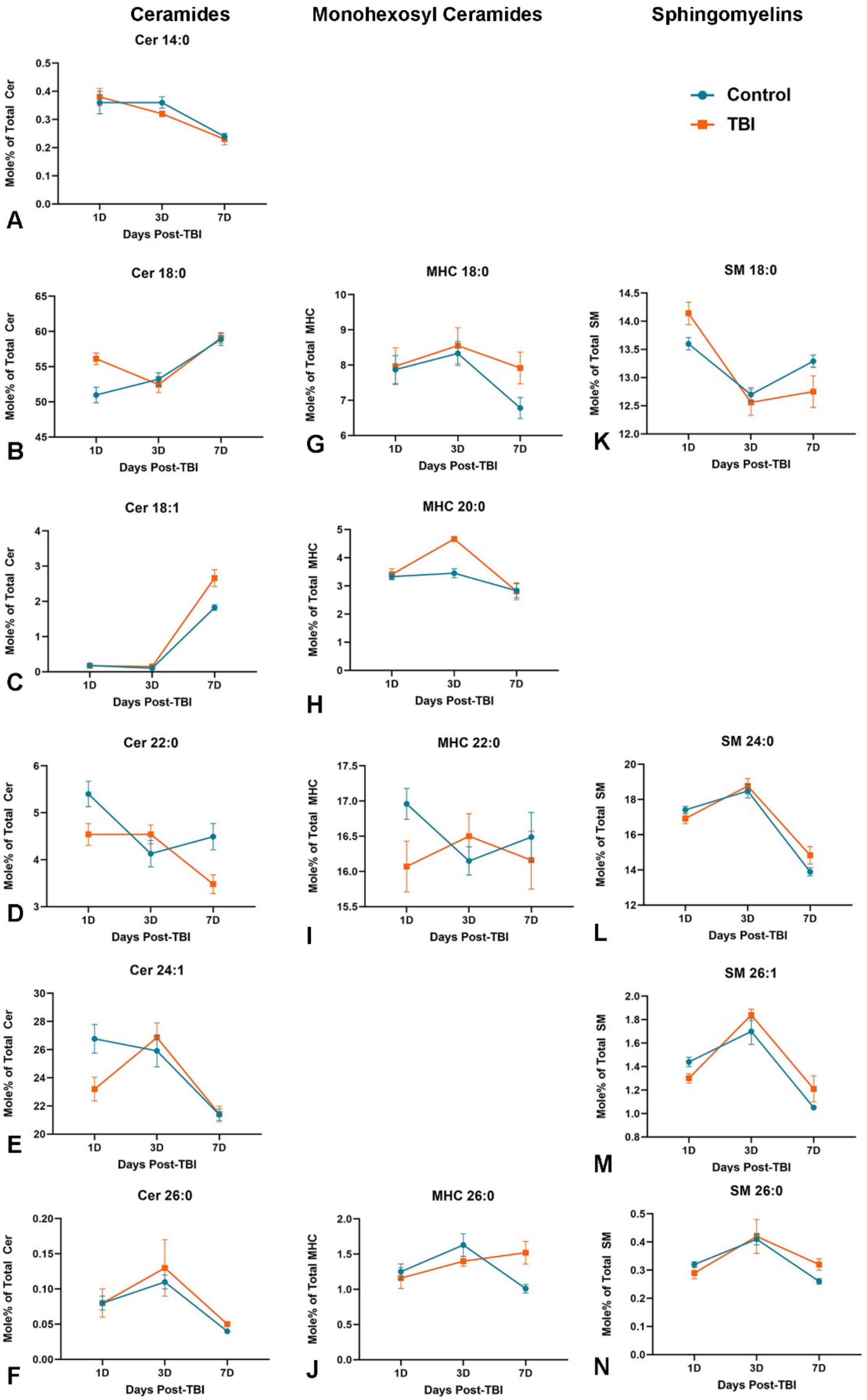

Figure S1. Longitudinal Variability of Brain Sphingolipid Species. Temporal changes in the levels of Ceramide (Cer), Monohexosylceramide (MHC), and Sphingomyelin (SM) species at 1 day, 3 days, and 7 days post-TBI.

Figure S2

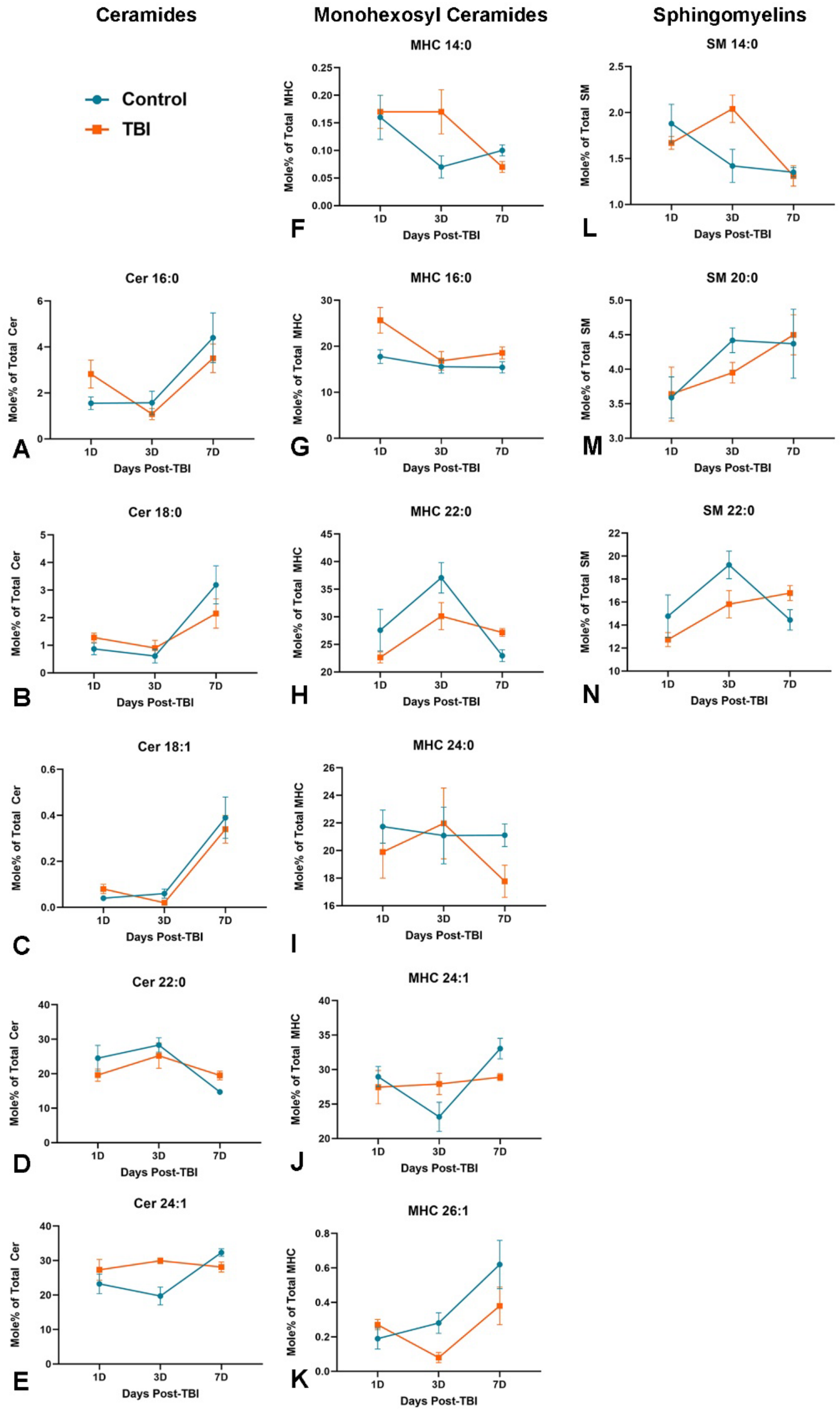

Figure S2. Longitudinal Variability of Plasma Sphingolipid Species.: Temporal changes in the levels of Ceramide (Cer), Monohexosylceramide (MHC), and Sphingomyelin (SM) species at 1 day, 3 days, and 7 days post-TBI.

Supplemental Table 1: Comparative Analysis of Significant Metabolite Changes in Brain and Plasma Following Traumatic Brain Injury (TBI).

**Species**	**Brain (Days Post-TBI)**	**Plasma (Days Post-TBI)**
Acid Sphingomyelinase (aSMase)	Elevation (1-3 days)	-
Neutral Sphingomyelinase (nSMase)	Elevation (7 days)	-
Ceramide-1-Phosphate (C1P)	Elevation (1 day)	-
Monohexosylceramide (MHC)	Elevation (7 days)	
Sphingosine	Elevation (7 days)	-
Cer C18:0	Elevation (1 day)	-
Cer C22:0	-	Decrease (1day), Increase (7 day)
Cer C24:0	-	Decrease (3 day)
Cer C24:1	Decrease (1 day)	Increase (3 days) Decrease (7 days)
MHC C16:0	-	Increase (1 day)
MHC C20:0	Elevation (3 days)	Decrease (1 and 3 day), Increase (7 days)
MHC C18:0	Decrease (7 days)	-
MHC C22:0	-	Decrease (1 day), decrease (3 days), increase (7 days)
MHC C24:0	-	Decrease (7 days)
MHC C24:1	Elevation (7 days)	Decrease (7 days)
SM C18:0	Elevation (7 days)	-
SM C22:0	-	Decrease (day 3), Increase (7 days)
SM C24:0	Elevation (7 days)	-
Expression of SPL Metabolic Genes	Increase (1 day), Reduction (3 days), Absence (7 days)	-
